# Rice endosperm is cost‐effective for the production of recombinant griffithsin with potent activity against HIV

**DOI:** 10.1111/pbi.12507

**Published:** 2016-01-23

**Authors:** Evangelia Vamvaka, Elsa Arcalis, Koreen Ramessar, Abbey Evans, Barry R. O'Keefe, Robin J. Shattock, Vicente Medina, Eva Stöger, Paul Christou, Teresa Capell

**Affiliations:** ^1^ Department of Plant Production and Forestry Science School of Agrifood and Forestry Science and Engineering (ETSEA) University of Lleida‐Agrotecnio Center Lleida Spain; ^2^ Department for Applied Genetics and Cell Biology Molecular Plant Physiology and Crop Biotechnology University of Natural Resources and Life Sciences Vienna Austria; ^3^ Molecular Targets Laboratory Center for Cancer Research National Cancer Institute NIH Frederick MD USA; ^4^ Department of Medicine Imperial College London, Norfolk Place London UK; ^5^ Natural Products Branch Developmental Therapeutics Program Division of Cancer Treatment and Diagnosis National Cancer Institute, NIH Frederick MD USA; ^6^ Catalan Institute for Research and Advanced Studies (ICREA) Barcelona Spain

**Keywords:** griffithsin, rice, endosperm, lectin, HIV, microbicides

## Abstract

Protein microbicides containing neutralizing antibodies and antiviral lectins may help to reduce the rate of infection with *human immunodeficiency virus* (HIV) if it is possible to manufacture the components in large quantities at a cost affordable in HIV‐endemic regions such as sub‐Saharan Africa. We expressed the antiviral lectin griffithsin (GRFT), which shows potent neutralizing activity against HIV, in the endosperm of transgenic rice plants (*Oryza sativa*), to determine whether rice can be used to produce inexpensive GRFT as a microbicide ingredient. The yield of ^OS^GRFT in the best‐performing plants was 223 μg/g dry seed weight. We also established a one‐step purification protocol, achieving a recovery of 74% and a purity of 80%, which potentially could be developed into a larger‐scale process to facilitate inexpensive downstream processing. ^OS^GRFT bound to HIV glycans with similar efficiency to GRFT produced in *Escherichia coli*. Whole‐cell assays using purified ^OS^GRFT and infectivity assays using crude extracts of transgenic rice endosperm confirmed that both crude and pure ^OS^GRFT showed potent activity against HIV and the crude extracts were not toxic towards human cell lines, suggesting they could be administered as a microbicide with only minimal processing. A freedom‐to‐operate analysis confirmed that GRFT produced in rice is suitable for commercial development, and an economic evaluation suggested that 1.8 kg/ha of pure GRFT could be produced from rice seeds. Our data therefore indicate that rice could be developed as an inexpensive production platform for GRFT as a microbicide component.

## Introduction

The rate of infection caused by *human immunodeficiency virus* (HIV) is declining, but the HIV^+^ population reached an estimated 35 million in 2013 and there were 2.1 million new cases (UNAIDS, 2014). The availability and effectiveness of antiretroviral therapy is also increasing, which means that the HIV^+^ population is living longer (Abdool Karim and Baxter, 2012; Lynch *et al*., 2012). However, the burden of the disease falls disproportionately on sub‐Saharan Africa, which accounts for more than 70% of global cases. Here, 1 in 20 people is HIV^+^ and up to 22 million have no access to life‐saving treatments (UNAIDS, 2014).

HIV infections cannot currently be cured, but there is a window of opportunity lasting 30–60 min between exposure and infection during which pre‐exposure prophylaxis can block the uptake of the virus (Shattock and Rosenberg, 2012). When the virus interacts with mucosal tissue, an infection is established within 16–72 h (Haase, 2011). Topical microbicides represent a subset of pre‐exposure prophylaxis strategies and are classified according to their target and mechanism of action. Surfactants kill the pathogen, replication inhibitors prevent virus replication, buffers enhance the vaginal milieu of protectors, and entry/fusion inhibitors block virus entry into host cells (McGowan, 2006).

Entry inhibitors can bind sites on the virus or its cellular receptors (CD4, CCR5 or CXCR4) to prevent virus–cell interactions. Some entry inhibitors are anionic polymers or polyanions, for example cellulose sulphate, PRO‐2000, dextrin sulphate, SPL7013, Carraguard, SAMMA and cellulose acetate phthalate. Although proven to be safe and well tolerated, these have failed to provide protection against HIV‐1 in clinical trials (El‐Sadr *et al*., 2006; Joshi *et al*., 2006; McCormack *et al*., 2005; Patton *et al*., 2006). Other more specific microbicides such as CCR5 inhibitors, for example PSC‐RANTES (Kawamura *et al*., 2003), and fusion inhibitors, such as the lectins griffithsin (GRFT) and cyanovirin‐N (CV‐N), demonstrate picomolar range IC_50_ values against all HIV‐1 clades but have yet to be tested in clinical trials (Ramessar *et al*., 2010).

GRFT is a 12.7 kDa lectin isolated from the red alga *Griffithsia* spp. (Mori *et al*., 2005). It inhibits HIV‐1 by binding selectively to mannose‐rich glycans on the virus envelope glycoproteins (Balzarini, 2005; Mori *et al*., 2005) and has demonstrated broad‐spectrum antiviral activity against *severe acute respiratory syndrome‐associated coronavirus* (SARS‐CoV) and other coronaviruses (O'Keefe *et al*., 2010; Ziółkowska *et al*., 2006), *hepatitis C virus* (Meuleman *et al*., 2011; Takebe *et al*., 2013), *Japanese encephalitis virus* (Ishag *et al*., 2013) and *herpes simplex virus* (Nixon *et al*., 2013). GRFT has no mitogenic effect on peripheral blood mononuclear cells, but shows full activity in the presence of macaque vaginal secretions (Emau *et al*., 2007) and has a good safety profile in the rabbit vaginal irritation model (O'Keefe *et al*., 2009). GRFT can also inhibit HIV‐1 binding to the dendritic cell‐specific intercellular adhesion molecule‐3‐grabbing nonintegrin (DC‐SIGN) and prevents DC‐SIGN‐mediated transfer of the virus to target cells (Alexandre *et al*., 2010).

Only small quantities of GRFT can be isolated from algae so the development of lectin‐based microbicides requires the expression of recombinant lectins in larger amounts. GRFT has been expressed as a recombinant protein in *Escherichia coli* (Giomarelli *et al*., 2006) and in *Nicotiana benthamiana* (O'Keefe *et al*., 2009) despite concerns that the production of recombinant lectins in plants might interfere with signalling processes and plant development (Lotter‐Stark *et al*., 2012). The yield of recombinant GRFT in *E. coli* (^EC^GRFT) was 819 mg per litre of culture medium but 33% of the protein accumulated as inclusion bodies and was therefore misfolded and nonfunctional (Giomarelli *et al*., 2006). In contrast, the transient expression of GRFT in *N. benthamiana* using a vector based on *tobacco mosaic virus* (TMV) resulted in the production of up to 1 g of soluble GRFT per kg of fresh leaf weight (O'Keefe *et al*., 2009). Even so, the transient expression of microbicides in leafy crops has several drawbacks, including the need to process the tissue immediately after harvest to prevent protein degradation, and the need for complete purification due to the presence of toxic metabolites in tobacco and endotoxins produced by the infiltrating bacteria (Arfi *et al*., 2015).

Transgenic seeds are advantageous because they can be stored for long periods under ambient conditions without recombinant proteins losing stability, and the generally regarded as safe (GRAS) status of cereal seeds means that microbicides could be administered as crude extracts rather than reformulated pure proteins, thus reducing production costs substantially (Peters and Stöger, 2011; Ramessar *et al*., 2008a; Sabalza *et al*., 2013). We therefore generated transgenic rice plants expressing GRFT in the endosperm and tested both the pure protein and crude extracts for their HIV‐specific binding activity and ability to neutralize HIV *in vitro*. Our studies were complemented with a griffithsin‐specific freedom‐to‐operate (FTO) analysis and a preliminary techno‐economic evaluation, which are essential first steps towards product development and commercialization.

## Results

### GRFT is expressed in T1 transgenic rice endosperm and is correctly folded in planta

**Figure 1:**
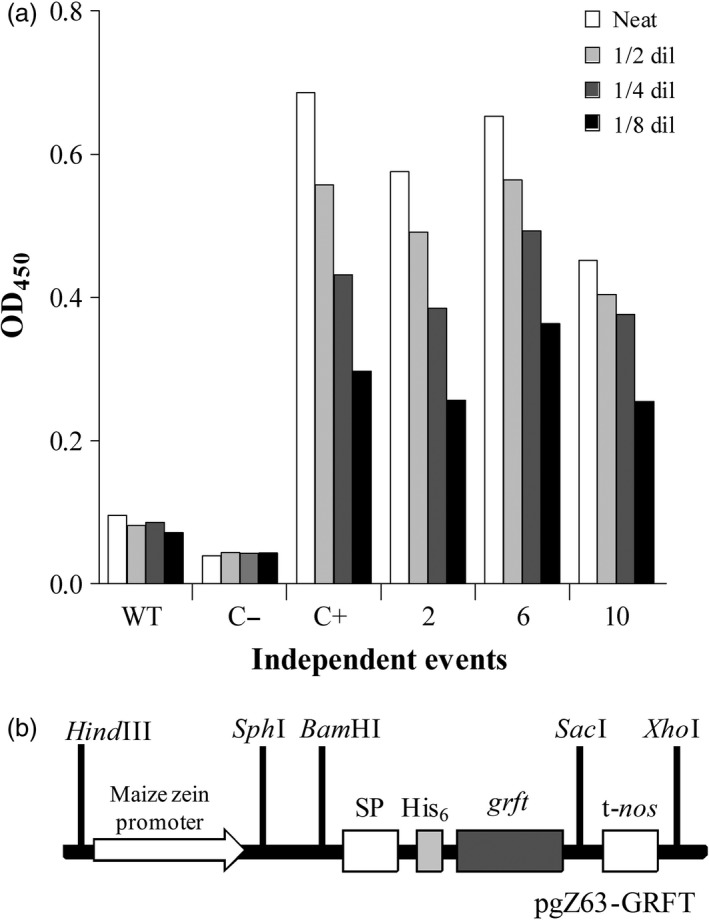
(a) GRFT accumulation in rice endosperm. An immunoglobulin‐specific sandwich ELISA was used to screen the three best‐performing independent events. The ELISA plate was coated with gp120, and GRFT protein assembly was confirmed using a primary rabbit anti‐GRFT polyclonal antiserum and a secondary HRP‐conjugated anti‐rabbit IgG antiserum. Four serial dilutions per sample are shown (neat, 1/2, 1/4 and 1/8). WT, wild‐type extracts; C^−^, negative control (PBS); C^+^, GRFT purified from *E. coli* (500 ng/mL) as a positive control. OD, optical density at 450 nm. (b) Transformation construct pgZ63‐GRFT for the stable expression of GRFT in rice endosperm. The expression cassette comprised the endosperm‐specific maize zein promoter, the rice α‐amylase 3A signal peptide sequence (SP), a His_6_ tag, the GRFT coding region (*grft*) and the *nos* terminator (t‐*nos*).

Mature seed‐derived rice embryos (15–20 days after pollination) were transformed by particle bombardment with a construct containing the *GRFT* coding sequence under the control of the maize zein promoter and a second construct containing the selectable marker *hpt* (Christou *et al*., 1991). Embryo‐derived callus was selected on hygromycin‐supplemented medium and 13 independent transformants were regenerated and transferred to the greenhouse. We chose three independent lines (2, 6 and 10) that expressed ^OS^GRFT at high levels in the endosperm (128, 301 and 104 μg/g dry seed weight, respectively). The yields were determined by enzyme‐linked immunosorbent assay (ELISA) to ensure that we detected only the correctly folded and soluble form of the protein. The remaining lines expressed ^OS^GRFT at only low levels and thus were deemed unsuitable for further experiments. The best‐performing line (line 6) was selected for further in‐depth investigation (Figure 1a).

### A one‐step protocol allows the inexpensive purification of ^OS^GRFT

**Figure 2:**
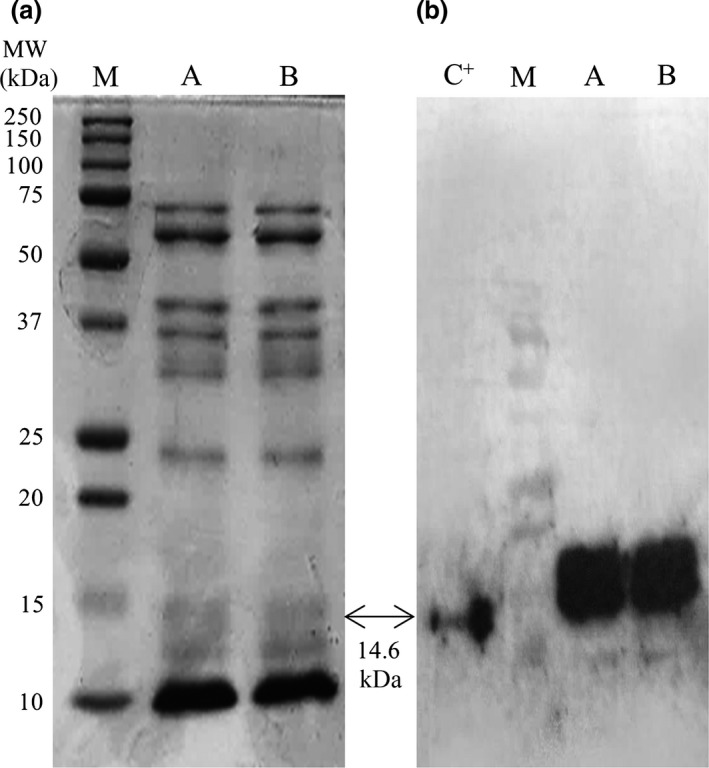
(a) Separation of the purified GRFT fraction by SDS‐PAGE under reducing conditions showing the 14.6 kDa band representing GRFT (arrow). M = Precision Plus Protein^™^ All Blue Standards (Bio‐Rad), lane A = 3.5 μg GRFT and lane B = 3.8 μg of GRFT protein. (b) Analysis of ^OS^GRFT by SDS‐PAGE under reducing conditions and immunoblotting using a primary rabbit anti‐GRFT polyclonal antiserum and a secondary HRP‐conjugated anti‐rabbit IgG. C^+^ = positive control (50 ng ^EC^GRFT), M = protein size marker Precision Plus Protein^™^ All Blue Standards (Bio‐Rad). The arrow shows the anticipated size of the GRFT‐His_6_ protein.

The ^OS^GRFT protein was purified from crude extracts of T1 rice endosperm prepared from line 6 by capturing the protein on an immobilized metal affinity chromatography (IMAC) column and eluting it with buffer containing 250 mm imidazole. The final yield of ^OS^GRFT after purification was 223 μg/g dry seed weight, which represents a recovery of 74%. The presence of ^OS^GRFT in the crude extracts was confirmed by reducing sodium dodecyl sulphate–polyacrylamide gel electrophoresis (SDS‐PAGE) followed by staining with Coomassie Brilliant Blue, showing a visible band of ~15 kDa, the anticipated molecular mass of His_6_‐tagged GRFT (Figure 2a). The presence of ^OS^GRFT was confirmed by immunoblotting and detection using a primary rabbit anti‐GRFT polyclonal antiserum (Figure 2b). This revealed an intense band at 14.6 kDa, the correct size for His_6_‐tagged GRFT, and another at 16–17 kDa probably representing the incomplete removal of the rice α‐amylase (RAmy3D) signal peptide which is 25 residues in length and has a predicted molecular mass of 2.6 kDa. Alternatively, the additional band may reflect O‐linked glycosylation at one of the multiple serine or threonine residues of GRFT. No degradation products were present, suggesting the protein remained stable in the rice endosperm tissue (Figure 2b).

### Crude rice endosperm extracts containing ^OS^GRFT show gp120‐binding activity comparable to pure ^EC^GRFT

**Figure 3:**
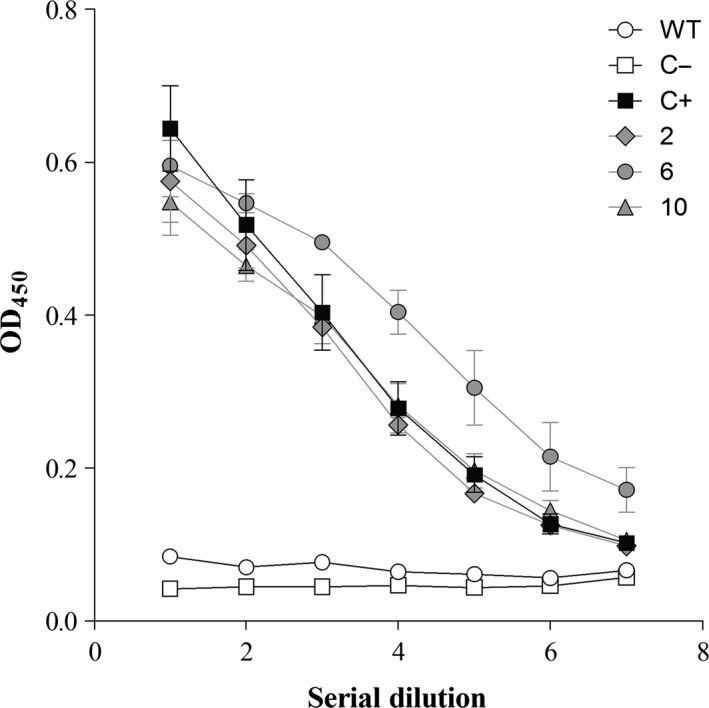
Antigen‐binding activity of crude rice endosperm extracts containing ^OS^GRFT and the purified ^EC^GRFT determined by ELISA. Ordinate: Optical density at 450 nm. Abscissa: Serial dilutions. Values are the average of two experiments ± SE. Plates coated with HIV‐1 gp120 were tested with different concentrations of crude extracts or fixed concentrations of purified GRFT from *E. coli,* and binding activity was measured using a primary rabbit anti‐GRFT polyclonal antiserum and a secondary HRP‐conjugated anti‐rabbit IgG. WT, wild‐type plants; C^−^, negative control (PBS); C^+^, ^EC^GRFT (concentration 500 ng/mL) as a positive control; 2, 6, 10, independent events expressing ^OS^GRFT; OD, optical density at 450 nm.

The crude extracts from T1 seeds of transgenic lines 2, 6 and 10 were tested by ELISA for *in vitro* binding activity against HIV gp120, using ^EC^GRFT as a positive control and wild‐type rice endosperm as a negative control. Extracts from all three transgenic lines showed greater gp120‐binding activity than the wild‐type endosperm extracts as expected, which confirmed that the ^OS^GRFT was functional and that endogenous plant lectins do not interfere with binding. Furthermore, crude endosperm extracts containing ^OS^GRFT bound to gp120 in a concentration‐dependent manner, with binding behaviour nearly identical to that of ^EC^GRFT. The crude extracts containing ^OS^GRFT therefore possessed essentially the same oligosaccharide‐dependent binding properties as the same recombinant protein expressed in bacteria (Figure 3).

### ^OS^GRFT accumulates to high levels in rice endosperm protein bodies

**Figure 4:**
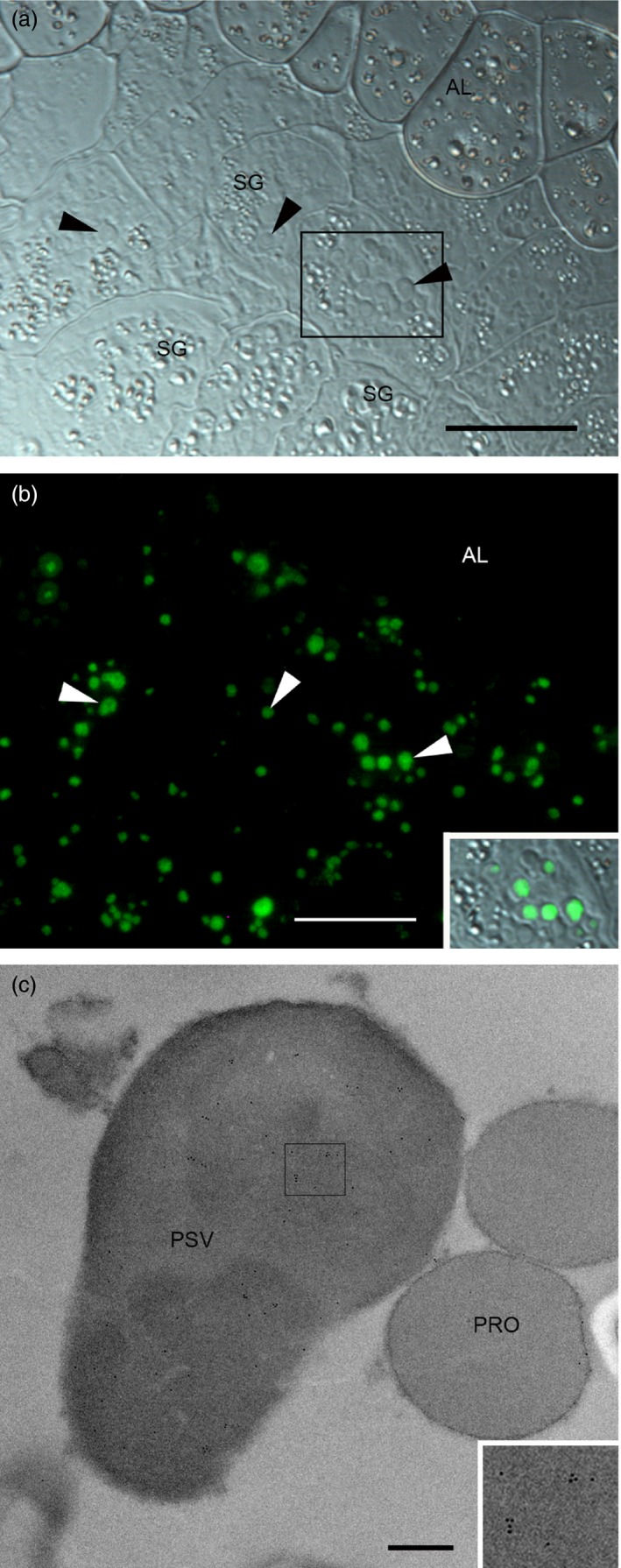
Localization of ^OS^GRFT in immature rice seeds, 15–20 days after pollination. (a) Interference contrast microscopy. (b) Fluorescence microscopy. The outlined area in (a) is shown as an inset panel in (b) with merged channels. (c) Immuno‐electron microscopy. Outlined area is shown enlarged twofold along each axis in the inset panel. Arrowheads in (a) and (b) represent storage organelles. Abbreviations: SG, starch granule; AL, aleurone layer; PRO, protein body; PSV, protein storage vacuole. Bars = 5 μm (a, b, including inset panel), 0.5 μm (c).

Fluorescence microscopy and transmission electron microscopy (TEM) were used to study the localization of ^OS^GRFT in the endosperm of developing seeds from line 6. The deposition of ^OS^GRFT was investigated in the young endosperm cells immediately under the aleurone layer, which are packed with starch grains, as well as storage organelles which show a strong labelling for GRFT (Figure 4a,b). No significant labelling was detected in any other compartment of the endosperm cells, the apoplast or the aleurone cells (Figure 4b). TEM imaging confirmed the deposition of GRFT in the protein storage vacuole (PSV), also known as type II protein bodies (PB‐II), whereas there was no significant labelling in the ER‐derived prolamin bodies, also known as type I protein bodies (PB‐I) (Figure 4c).

### Crude extracts containing ^OS^GRFT and purified ^OS^GRFT show potent neutralizing activity against HIV in whole‐cell and infectivity assays but no toxicity towards human cells

**Figure 5:**
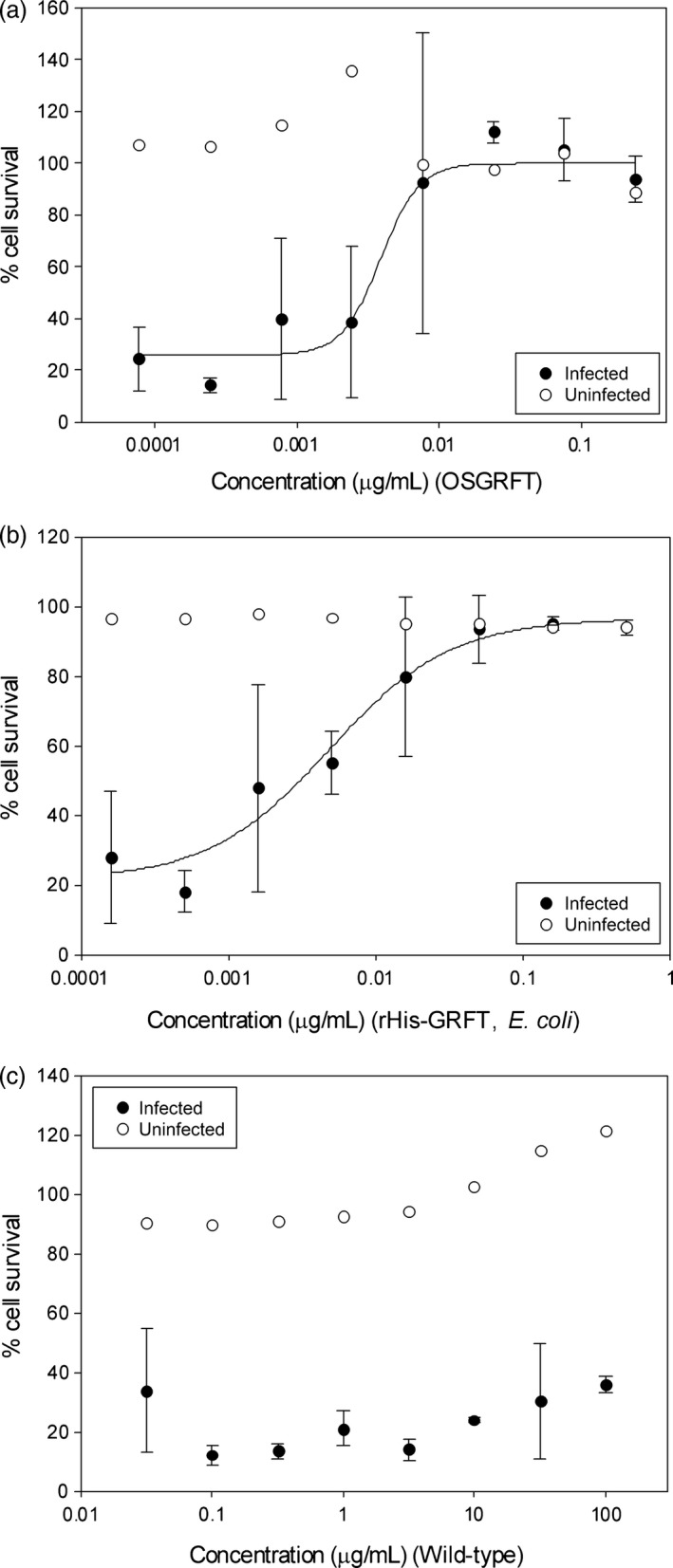
Concentration‐dependent effects of GRFT on cellular viability. The *in vitro* HIV‐neutralizing activity of (a) ^OS^GRFT, (b) ^ES^GRFT and (c) wild‐type rice extracts against CEM‐SS cells infected with HIV‐1_RF_ is presented as cell viability relative to uninfected and untreated controls. Cell viability was assessed using the XTT assay. All points are averages (±SE) of triplicate measurements.

**Figure 6:**
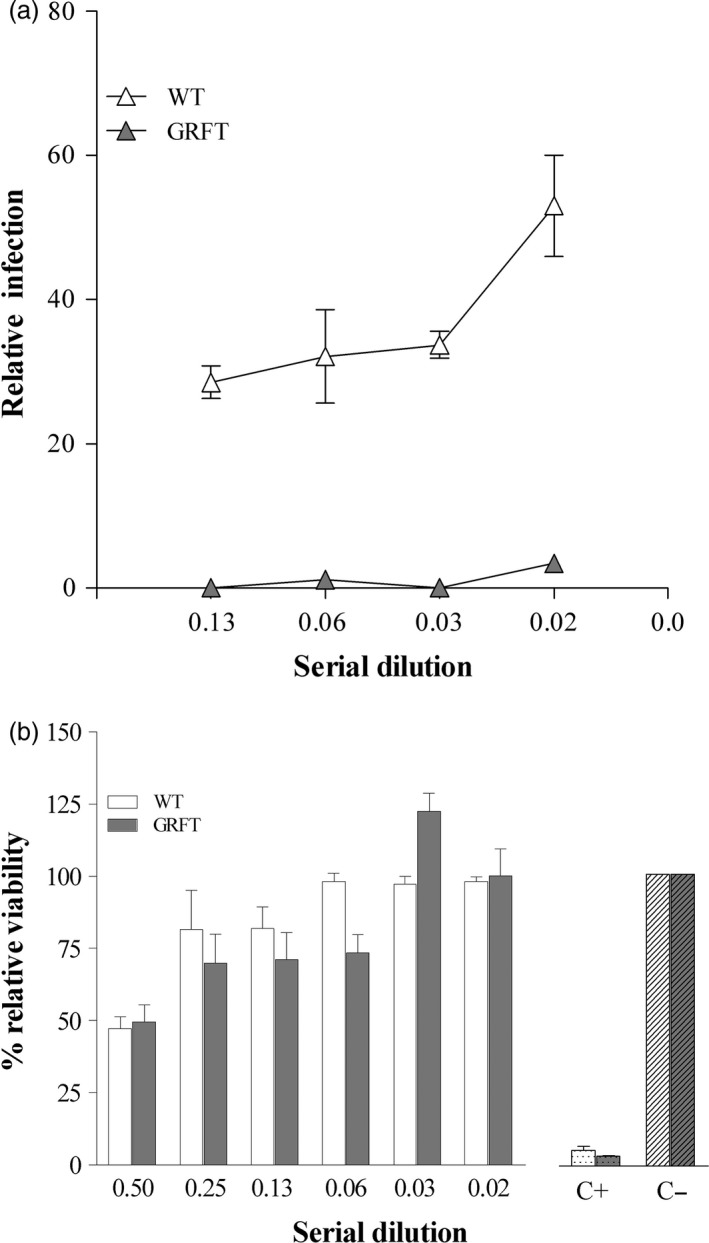
(a) HIV‐1 infectivity assay. TZM‐bl cells were incubated with serial dilutions of the extracts (in triplicate) and cultured for 72 h with HIV‐1BaL using a concentration optimized for infectivity. The extent of viral replication was determined by measuring the luciferase activity of cell lysates. Ordinate: Percentage of relative infection. Abscissa: Serial dilutions. Results are shown as the percentage of infection relative to the virus‐only control ± SE. (b) Effect of different concentrations of crude extracts on cell viability. Cytotoxicity was determined using an MTT assay and was expressed as the percentage of dead cells (mean ± SD, *n* = 3). Ordinate: Percentage relative viability. Abscissa: Serial dilutions. WT, untransformed rice seed extract; C^−^, negative (medium‐only) control; C^+^, cytotoxic positive control (nonoxynol‐9).

We next tested purified ^OS^GRFT and ^EC^GRFT head to head in whole‐cell HIV neutralization assays. As expected from their similar *in vitro* binding behaviour, both proteins achieved similar EC_50_ values in the nanomolar range: 0.27 nm for ^OS^GRFT (Figure 5a) and 0.33 nm for ^EC^GRFT (Figure 5b). The wild‐type crude extract showed no activity against HIV in the whole‐cell assay, although it did cause a general increase in cell growth in both controls and virus‐challenged cells in culture, confirming that native rice lectins do not possess HIV‐neutralizing activity (Figure 5c). Infectivity assays confirmed the potent antiviral activity of crude extracts containing ^OS^GRFT. Crude extracts of wild‐type rice endosperm also showed a low level of viral inhibition, perhaps due to the presence of a protein/polysaccharide milieu that delays contact between the virus and the target cells (Figure 6a). Crude extracts of rice endosperm containing ^OS^GRFT at dilutions of 1/4 and higher showed no significant cytotoxicity against the cell line, indicating that microbicides prepared with crude extracts are unlikely to be harmful when used for topical application (Figure 6b).

## Discussion

Griffithsin (GRFT) is a lectin isolated from the red alga *Griffithsia* spp. that binds to high mannose oligosaccharides displayed on the envelope glycoproteins of many different viruses (Xue *et al*., 2013) including HIV (Ziółkowska *et al*., 2007a). GRFT is active against HIV clades A, B and C, which are predominant in sub‐Saharan Africa, India and the west (Lotter‐Stark *et al*., 2012), and is equally active against T‐tropic and M‐tropic strains of HIV‐1 (Mori *et al*., 2005). GRFT acts as an HIV entry inhibitor, blocking virus–cell fusion at subnanomolar concentrations and reducing the cytopathic effect of laboratory strains and clinical primary isolates of HIV‐1 at picomolar concentrations (Mori *et al*., 2005).

Native GRFT can be purified directly from its source but only in minute quantities (Mori *et al*., 2005). The production of recombinant GRFT is therefore necessary to meet the demand for this protein as a microbicide component. GRFT has been produced in *E. coli* with a yield of 819 mg/l, but only 66% of the protein was present in the soluble fraction and the remainder was found in inclusion bodies and was impossible to resolubilize even using detergents (Giomarelli *et al*., 2006). O'Keefe *et al*. (2009) addressed this challenge by producing soluble GRFT in *N. benthamiana* leaves using a vector based on TMV, achieving a yield of 1 mg/g leaf tissue, which was reduced to 0.3 mg/g after purification, representing a recovery of 30%.

We investigated the use of rice endosperm as an alternative production platform for recombinant GRFT because rice has a number of application‐specific advantages over leafy crops in the context of microbicides. Like other seed‐based platforms, the recombinant protein accumulates in dry seeds and can be stored indefinitely under ambient conditions, whereas tobacco leaves must be processed immediately after harvesting to avoid protein degradation (Ramessar *et al*., 2008a; Sabalza *et al*., 2013). Furthermore, rice has GRAS status and has been developed as a production platform for proteins that can be administered in minimally processed seed, such as oral vaccines and prophylactics (An *et al*., 2013; He *et al*., 2011; Ning *et al*., 2008; Vamvaka *et al*., 2015; Xie *et al*., 2008; Zhang *et al*., 2012), whereas proteins transiently expressed in tobacco must be purified to remove toxic metabolites (Obembe *et al*., 2011) and the bacterial endotoxins produced during agroinfiltration (Arfi *et al*., 2015).

We created transgenic rice plants expressing GRFT under the control of an endosperm‐specific promoter and recovered 13 independent transgenic events, three of which were selected for further investigation because they produced T1 seeds in numbers sufficient for analysis. This is the first time that GRFT has been expressed in stably transformed plants and the first time it has been expressed in cereal seeds. Line 6 was selected for additional experiments because T1 seeds produced the highest yields of ^OS^GRFT (301 μg/g dry seed weight). GRFT has been produced at higher levels by transient expression in *N. benthamiana* (1 mg/g after 12 days), but this was reduced to 300 μg/g after purification, which is only slightly higher than our yields of pure protein (223 μg/g dry seed weight) and similar to the yield of 350 μg/g reported for another lectin (CV‐N) produced in transgenic soybean (O᾽Keefe *et al*., 2015). It is also likely that our T1 yields can be improved further by breeding line 6 to homozygosity, as demonstrated for other recombinant proteins produced in cereal seeds (Hood *et al*., 2012). For example, the monoclonal antibody 2G12 was expressed in rice seeds with a yield of 37 μg/g in the T1 generation but this increased to 46 μg/g in the T3 generation after two rounds of self‐pollination (Vamvaka *et al*., 2015). Furthermore, 2G12 was expressed in maize seeds with a yield of 75 μg/g, which was increased to 100 μg/g by passaging the transgenic plants through a dedifferentiation–differentiation cycle (Ramessar *et al*., 2008b).

The number and nature of purification steps and the overall cost of processing are key issues that determine the commercial viability of recombinant proteins expressed in plants. Mori *et al*. (2005) purified the native GRFT from *Griffithsia* spp. by ammonium sulphate precipitation, hydrophobic interaction chromatography, anion‐exchange chromatography, reversed‐phase chromatography and size exclusion chromatography to yield a homogeneous, highly pure and biologically active protein. We found that ammonium sulphate precipitation did not work for ^OS^GRFT even when combined with ethanol precipitation. However, protein extraction using a phosphate buffer combined with IMAC achieved 74% recovery and 80% purity. Giomarelli *et al*. (2006) purified soluble ^EC^GRFT by IMAC using Ni‐NTA resin and the dialysed protein was then applied to a TALON IMAC resin before stepwise elution using two buffers containing different amounts of imidazole, achieving 53% recovery. Ishag *et al*. (2013) used IMAC followed by elution in a buffer containing 250 mm imidazole before further purification by size exclusion chromatography using a Superdex 75 column. The resulting purified protein contained both the monomeric and dimeric forms of GRFT but the final purity and recovery were not reported. Finally, Xue *et al*. (2013) used a nickel‐chelating column and eluted GRFT with 500 mm imidazole, refolding the protein by dropwise addition to a low‐salt refolding buffer before final purification using C4 reversed‐phase chromatography, but again no data were reported for purity and recovery. When GRFT was produced in *N. benthamiana*, the first purification step involved the removal of the TMV vector coat protein by filtration through a ceramic membrane, followed by ion‐exchange chromatography over SP‐Sepharose resin, ultrafiltration/diafiltration to concentrate the protein and elution with 100 mm NaCl, achieving >99.8% purity albeit with only 30% recovery as stated above (O'Keefe *et al*., 2009). We used nondenaturing conditions to prepare the protein extract thus keeping GRFT in its native confirmation to avoid the need for a refolding step. The protein was captured on a Profinity IMAC column and eluted with 250 mm imidazole. This is the first one‐step purification procedure reported for GRFT, and although the purity was lower than that reported by O'Keefe *et al*. (2009), the use of rice as a production platform means that stringent purity is unnecessary because the plant matrix is safe for mucosal applications (Ning *et al*., 2008; Xie *et al*., 2008). The large‐scale production and purification of GRFT could be facilitated by exploiting its stability, because it remains soluble and functional at temperatures up to ~80 °C, whereas most plant host cell proteins precipitate below this temperature (Moulaei *et al*., 2010; Ziółkowska *et al*., 2007b). Fuqua *et al*. (2015) demonstrated that the purity of GRFT extracts can increase by 30%–40% at temperatures exceeding 50 °C, and using this approach, they achieved a recovery rate of 88 ± 5% compared to the initial extract.

Immunoblotting using a primary rabbit anti‐GRFT polyclonal antiserum revealed a pair of intense bands, one migrating at the anticipated molecular mass of 14.6 kDa and the other at 16–17 kDa. Because the gel was run under denaturing conditions, the additional band is unlikely to represent a conformational isomer or the presence of a noncovalent binding partner. It is possible that GRFT undergoes partial post‐translational modification and is therefore present in two forms, but the GRFT sequence does not contain an N‐glycan acceptor site so only O‐linked glycans would account for the difference in mobility (2–3 kDa). A more likely explanation is the incomplete removal of the rice α‐amylase (RAmy3D) signal peptide, which is 25 residues in length and has a predicted molecular mass of 2.6 kDa.

The deposition of GRFT in rice endosperm was studied by fluorescence and electron microscopy. Our data confirmed that GRFT is deposited predominantly in the PSVs, like other recombinant proteins expressed in cereal endosperm bearing a signal peptide but no further targeting information. For example, a recombinant fungal phytase was directed to the PSVs in rice and wheat, and the HIV‐neutralizing antibody 2G12 was deposited in the PSVs of maize and rice (Arcalis *et al*., 2004; Drakakaki *et al*., 2006; Peters *et al*., 2013; Vamvaka *et al*., 2015). Furthermore, Drakakaki *et al*. (2006) described the presence of recombinant phytase in ER‐derived protein bodies, and the expression of antibody 2G12 altered the morphology of zein bodies in maize and induced the formation of novel ER‐derived protein bodies in rice (Peters *et al*., 2013; Vamvaka *et al*., 2015). In contrast, we found that GRFT was localized almost exclusively in the PSVs and could easily be extracted with PBS even though most of the surrounding endogenous seed storage proteins are not soluble under these conditions. The ability of GRFT to retain its independent solubility suggests that it does not interact with the storage proteins, for example by the formation of disulphide bonds (Peters *et al*., 2013).

GRFT can interfere with the interaction between HIV and CD4^+^ cells by binding to the mannose‐rich viral glycoproteins gp120, gp41 and gp160, although it does not directly inhibit the binding of gp120 to CD4 (Mori *et al*., 2005). GRFT interacts with a number of viruses, but shows particular potency against HIV because it can bind to multiple sites on gp120 (Giomarelli *et al*., 2006). We therefore compared the biological activity of purified ^OS^GRFT, crude extracts containing ^OS^GRFT and purified ^EC^GRFT, by testing their ability to bind gp120‐IIIB *in vitro*. We found that ^OS^GRFT and ^EC^GRFT bound to gp120 with near identical binding characteristics and that the crude extracts showed similar binding activity to the pure proteins once the concentration of GRFT in the extracts was taken into account. Similarly, O'Keefe *et al*. (2009) showed that *N. benthamiana* GRFT had similar or even better gp120‐binding activity than ^EC^GRFT, which in turn retained the gp120‐binding activity of the native GRFT protein (Giomarelli *et al*., 2002).

We also tested ^OS^GRFT and ^EC^GRFT head to head in whole‐cell HIV neutralization assays and found that both proteins achieved near identical nanomolar EC_50_ values of 0.27 and 0.33 nm, respectively, well within the range previously reported by Mori *et al*. (2005). Crude extracts of wild‐type rice endosperm showed no activity against HIV. Giomarelli *et al*. (2006) showed that ^EC^GRFT inhibited HIV‐induced cytopathicity with an EC_50_ value of 0.089 nm, similar to the EC_50_ value of native GRFT (0.163 nm) and they concluded that both forms of GRFT were functionally equivalent. However, O'Keefe *et al*. (2009) reported an EC_50_ value of 0.054 nm for native GRFT and 0.156 nm for tobacco‐derived GRFT. These discrepancies reflect the inherent variability of the syncytium inhibition assay, which is suitable for comparisons within an experiment (where 2‐ to threefold differences are not regarded as significant) but not across studies in different laboratories, particularly when different HIV strains and assay protocols are used (Forthal *et al*., 2010; Rademacher *et al*., 2008).

Candidate microbicides must be tested for immunogenicity, efficacy and safety before deployment, and *in vitro* cellular models can be used to provide preliminary evidence for these endpoints before progressing to *ex vivo* mucosal tissue explants, small animal models and nonhuman primates. The evaluation of potential anti‐HIV microbicides must consider the possibility of immune cell stimulation, that is when producing a new version of GRFT, it is important to rule out the ability of variants, process contaminants and other impurities to provoke an immune response. We found that ^OS^GRFT did not stimulate the proliferation of the CEM‐SS T‐lymphocytic cell line used in the cytotoxicity assays (Figure 5a) which at this stage provides confidence that the product does not cause immune cell activation, although further more detailed studies involving the analysis of cytokine levels would be required during preclinical development.

There are several cell lines that are widely used to test the efficacy of HIV microbicides, including PM‐1 CD4^+^T cells (Lusso *et al*., 1995) and the TZM‐bl luciferase reporter cell line (Wei *et al*., 2002). We carried out infectivity assays using TZM‐bl cells, which confirmed the potent activity of rice crude extracts containing ^OS^GRFT against HIV‐1 and achieved almost 100% prevention of infection. In terms of safety, cellular models are used to assess the potential cytotoxicity of microbicides, often by measuring the cleavage of a tetrazolium salt (MTT) into a blue‐coloured product (formazan) in viable cells (Slater *et al*., 1963). We found that crude rice endosperm extracts at dilutions of 1/4 and higher showed no significant cytotoxicity against the TZM‐bl HeLa cervical epithelium cell line, suggesting that microbicides based on crude rice endosperm extracts are unlikely to cause cytotoxic effects when applied to mucosal surfaces. This result confirms earlier studies with *N. benthamiana*‐produced GRFT, which confirmed its excellent safety profile (Kouokam *et al*., 2011).

A commercially viable large‐scale production platform for GRFT must achieve adequate yields to meet demand, including the recovery rate after purification, if relevant. The recovery of recombinant proteins purified from rice can range from 1.8% recovery with 95% purity to 55.8% recovery with more than 99.5% purity (Ou *et al*., 2014). Where high purity is not a major requirement (i.e. when using crude extracts for topical application), then the overall productivity of a rice production platform can be calculated based on the techno‐economic evaluation presented by Nandi *et al*. (2002). Assuming grain yields of up to 8 ton/ha, at least 1.8 kg of ^OS^GRFT could be harvested per hectare, which is 4.5‐fold higher than the ~0.4 kg/ha of GRFT possible in *N. benthamiana* (O'Keefe *et al*., 2009) but lower than 6.6 kg/ha of pure CV‐N produced in soybean seeds (O᾽Keefe *et al*., 2015). Kaufman and Kalaitzandonakes (2011) have shown that in order to scale up production to meet a demand of 1000 kg of purified recombinant protein per year, at least 550 ha would be required assuming no losses, and nearly 950 ha would be required to accommodate production risks of 20% and an anticipated recovery rate of 75%.

The broad utility of ^OS^GRFT in countries suffering the greatest burden of HIV would also benefit from the absence of intellectual property constraints. FTO analysis based on the simplified procedure described by Miralpeix *et al*. (2014) revealed four key patents covering GRFT‐related technologies, two of which potentially affect the commercial development of ^OS^GRFT, whereas the third is for chimeric proteins, protein combinations and other compositions, and the fourth is limited to monomeric GRFT and therefore does not affect the development of multimeric forms of the protein (Boyd *et al*., 2011; LiWang and Kagiampakis, 2011; O'Keefe *et al*., 2011, 2014). The principal claims made in these patents are summarized in Table S1. Although we have demonstrated that rice is potentially a cost‐effective production platform for GRFT, the FTO analysis revealed a possibility of patent infringement based on the GRFT sequence. However, the United States National Institutes of Health (NIH), which holds the most pertinent GRFT patents (Boyd *et al*., 2011, 2011; O'Keefe *et al*., 2011), has made the conscientious decision not to patent GRFT‐related technologies in developing countries so as to allow these countries, many of them those most affected by the HIV epidemic, to produce GRFT for use by their populations without a licence. This strategy will make ^OS^GRFT available royalty free in developing countries (where it is needed most), whereas conventional licensing options would be required in the developed world (Kryder *et al*., 2000).

In conclusion, our data show that GRFT can be expressed in rice endosperm with yields comparable to or higher than GRFT produced by transient expression in *N. benthamiana*. Both the crude extracts and the purified ^OS^GRFT showed near identical *in vitro* binding and HIV neutralization activity to ^EC^GRFT, confirming that ^OS^GRFT is correctly folded *in planta* and remains biologically active in the crude extracts and following purification. The functionality of the crude and purified protein together with the absence of cytotoxicity shows that rice could be developed as a cost‐effective production platform with minimal processing or one‐step purification strategies, thus offering the prospect of inexpensive microbicides suitable for deployment in HIV‐endemic areas. Preliminary FTO and techno‐economic analysis suggests that ^OS^GRFT could be commercially feasible using the humanitarian licensing strategy adopted by the NIH for developing country applications and conventional licensing elsewhere.

## Experimental procedures

### Expression construct

The *GRFT* gene in vector pET‐28a(+) was amplified by PCR in a 50 μL reaction containing 1.25 units of GoTaq polymerase in the appropriate buffer (Promega, Madison, WI), 1 μm each of forward primer 5′‐TGC ATG CAT GGG CAG CAG CCA TCA T‐3′ and reverse primer 5′‐GGG GAG CTC TTA GTA CTG TTC ATA GTA G‐3′ (the underlined nucleotides are SphI and SacI restriction sites introduced to facilitate cloning), 0.2 mm of each dNTP and 250 ng template DNA. The reaction was heated to 94 °C for 3 min followed by 35 cycles of 94 °C for 45 s, 60 °C for 45 s and 72 °C for 3 min, followed by a final extension step at 72 °C for 10 min. The products were transferred to the shuttle vector pGEM‐T Easy (Promega) and introduced into competent *E. coli* cells, which were incubated overnight at 37 °C under ampicillin selection. The integrity of the plasmid DNA was confirmed by sequencing (Universidad Autónoma de Barcelona, Spain) before digestion with SphI and SacI to release the expression cassette, which was then inserted into vector pgZ63 containing the endosperm‐specific maize zein promoter (Naqvi *et al*., 2009), the rice α‐amylase 3A signal peptide sequence (GenBank CAA39776) and a sequence encoding a N‐terminal His_6_ affinity tag (Figure 1b).

### Transformation and recovery of transgenic plants

Seven‐day‐old mature rice zygotic embryos (*Oryza sativa* cv. Nipponbare) were transferred to osmotic medium (4.4 g/l Murashige & Skoog (MS) powder supplemented with 0.3 g/l casein hydrolysate, 0.5 g/l proline, 72.8 g/l mannitol and 30 g/l sucrose) 4 h before bombardment with 10 mg gold particles coated with the *GRFT* construct and the selectable marker *hpt* at a 3 : 1 ratio (Christou *et al*., 1991; Sudhakar *et al*., 1998; Valdez *et al*., 1998). The embryos were returned to osmotic medium for 12 h before selection on MS medium (4.4 g/l MS powder, 0.3 g/l casein, 0.5 g/l proline and 30 g/l sucrose) supplemented with 50 mg/l hygromycin and 2.5 mg/l 2,4‐dichlorophenoxyacetic acid in the dark for 2–3 weeks. Transgenic plantlets were regenerated and hardened off in soil. Plants were grown in the greenhouse or growth chamber at 28/20 °C day/night temperature with a 10‐h photoperiod and 60%–90% relative humidity for the first 50 days, followed by maintenance at 21/18 °C day/night temperature with a 16‐h photoperiod thereafter in a growth chamber.

### Enzyme‐linked immunosorbent assay

The accumulation of GRFT in transgenic endosperm tissue was confirmed by ELISA. Mature rice seeds were ground in three volumes of PBS and centrifuged twice at 13 000 *g* for 10 min at 4 °C to remove debris. GRFT accumulation was confirmed by coating the wells of ELISA plates with 100 ng recombinant gp120 from HIV‐1 strain IIIB, provided by the MRC Centralized Facility for AIDS Reagents, Potters Bar, UK. After washing and blocking with 2.5% bovine serum albumin (BSA) in PBS containing 0.1% Tween‐20 (PBST), serial dilutions of each seed extract were added and protein assembly was confirmed using a primary rabbit anti‐GRFT polyclonal antiserum (The Binding Site, Birmingham, UK) and a secondary horseradish peroxidase (HRP)‐conjugated anti‐rabbit IgG antibody (The Binding Site), each diluted 1 : 1000. HRP was detected by adding the substrate 3,3′,5,5′‐tetramethylbenzidine (TMB) (Sigma‐Aldrich, St. Louis, MO) and reading the absorbance at 450 nm.

### Purification by affinity chromatography

Mature rice seeds were ground to a fine powder and extracted overnight at 4 °C in five volumes of lysis buffer (50 mm NaH_2_PO_4_, 300 mm NaCl, 5 mm imidazole, pH 8). Insoluble material was removed by centrifuging twice at 8000 *g* for 30 min at 4 °C. Each sample was loaded onto a Profinity IMAC column at a flow rate of 2 mL/min. The column was washed twice with wash buffer (50 mm NaH_2_PO_4_, 300 mm NaCl, 10 mm imidazole, pH 8) and the protein was eluted four times with elution buffer (50 mm NaH_2_PO_4_, 300 mm NaCl, 250 mm imidazole, pH 8). Protein‐containing fractions were identified using a NanoDrop 2000c UV‐Vis spectrophotometer (Thermo Fisher Scientific, Waltham, MA) based on extinction coefficient at 280 nm of the protein, and GRFT concentrations were determined by ELISA (see above). Fractions containing >50 μg/mL GRFT were pooled and concentrated by ultrafiltration using spin columns with a 3 kDa molecular weight cut‐off (Amicon Ultra, EMD Millipore, Darmstadt, Germany).

### SDS‐PAGE and immunoblotting

The efficiency of protein purification was tested by SDS‐PAGE in 4–12% precast Bis‐Tris NuPAGE gels (Invitrogen, Carlsbad, CA) using Precision Plus Protein Standards (Bio‐Rad, Hercules). Each sample comprised 5 μL of rice seed extract and 5 μL SDS loading buffer (0.3 m Tris‐HCl pH 6.8, 10% (w/v) SDS, 50% (v/v) glycerol, 0.125% (w/v) bromophenol blue). For immunoblotting, samples were transferred to nitrocellulose membranes using the Hoefer TE70 semidry transfer system (Amersham Biosciences, Piscataway, NJ) and blocked with 5% nonfat dried milk in Tris‐buffered saline (TBS) (50 mm Tris, 150 mm NaCl, pH 7.6). After three washes with TBS containing 0.1% Tween‐20 (TBST), protein expression was confirmed using the primary rabbit anti‐GRFT polyclonal antiserum and a secondary HRP‐conjugated anti‐rabbit IgG antiserum described above, each diluted 1 : 1000. The signal was detected using the ECL Plus Western Blotting Detection System (GE Healthcare, Amersham, Little Chalfont, UK).

### *In vitro* gp120‐binding assay

The specific antigen‐binding activity of GRFT was determined by coating the wells of ELISA plates with 100 ng recombinant gp120 from HIV‐1 strain IIIB, provided by the MRC Centralized Facility for AIDS Reagents, Potters Bar, UK. After washing with PBST and blocking with 2.5% BSA in PBST, serial dilutions of the GRFT protein were added and the amount of bound protein determined using a primary rabbit anti‐GRFT polyclonal antiserum and secondary HRP‐conjugated anti‐rabbit IgG antiserum diluted 1 : 1000. The signal was developed with TMB substrate and the absorbance was read at 450 nm.

### Microscopy

Immature rice seeds (15–20 days after pollination) from wild‐type plants and transgenic line 6 were fixed and processed for microscopy as previously described (Arcalis *et al*., 2004). Briefly, 1 mm^3^ seed fragments were fixed in 4% (w/v) paraformaldehyde and 0.5% (v/v) glutaraldehyde in 0.1 M phosphate buffer (pH 7.4) overnight at 4 °C, then dehydrated through an ethanol series and infiltrated in LR White resin before polymerization at 60 °C. We prepared 1‐μm sections for fluorescence microscopy, and ultrathin sections showing silver interference were used for TEM. The sections were incubated with a polyclonal rabbit anti‐GRFT antibody and secondary antibodies labelled with Alexa Fluor^®^ 488 for fluorescence microscopy or 10‐nm gold particles for electron microscopy. The sections were analysed on a Leica DM5500B fluorescence microscope and a FEI Tecnai G2 electron microscope operating at 160 kV.

### Whole‐cell HIV neutralization assays

Whole‐cell HIV neutralization assays were carried out using the 2,3‐bis‐[2‐methoxy‐4‐nitro‐5‐sulfophenyl]‐2H‐tetrazolium‐5‐carbox‐anilide inner salt (XTT) tetrazolium system. The HIV‐neutralizing activities of ^EC^GRFT and ^OS^GRFT were compared in CEM‐SS cells challenged with HIV‐1_RF_ (Gulakowski *et al*., 1991). XTT was provided by the Drug Synthesis and Chemistry Branch, Developmental Therapeutics Program, Division of Cancer Treatment and Diagnosis, National Cancer Institute. CEM‐SS cells were maintained in RPMI 1640 medium without phenol red and supplemented with 5% foetal bovine serum, 2 mm l‐glutamine and 50 μg/mL gentamicin (BioWhittaker). Exponentially growing cells were washed and resuspended in complete medium, and a 50‐μL aliquot containing 5 × 10^3^ cells was added to individual wells of a 96‐well round‐bottomed microtiter plate containing serial dilutions of ^EC^GRFT or ^OS^GRFT in 100 μL medium. Stock supernatants of HIV‐1_RF_ were diluted in complete medium to yield sufficient cytopathicity (80%–90% cell death in 6 days), and a 50‐μL aliquot was added to the appropriate wells. Additional control wells did not receive viral challenge so as to measure any compound‐induced effect on cell growth. Plates were incubated for 6 days at 37 °C and then stained with XTT to detect viable cells. The resulting data display both the protection of cells from the cytopathic effects of HIV‐1 and any direct cytotoxic effects of the test compounds on CEM‐SS cells. All experiments were carried out in triplicate.

### Cell and virus culture

TZM‐bl cells (NIH AIDS Reagent Program), a HeLa cell line expressing CD4, CCR5 and luciferase (Platt *et al*., 2009), were grown in continual culture in Dulbecco's modified Eagle medium (DMEM) supplemented with 10% foetal calf serum, 100 U/mL penicillin, 100 μg/mL streptomycin and 2 mm l‐glutamine. HIV‐1BaL (R5) was grown in PM‐1 CD4^+^ T cells. Cell free viral stocks were passed through 0.2‐μm filters.

### Cytotoxicity and antiviral assays

The viability of cells following exposure to rice seed crude extracts as well as the cytotoxicity of the crude extracts was assessed using the MTT colorimetric assay as previously described (Mesquita *et al*., 2008). Crude extracts of wild‐type and transgenic rice endosperm expressing ^OS^GRFT were diluted to 50% concentration; then, a twofold serial dilution was performed. TZM‐bl cells (10^5^/well) were cultured in the presence of extracts for 24 h at 37 °C and titrated in triplicate onto 10^5^ TZM‐bl cells per well. Twofold serial dilutions of the extracts were prepared for testing, and nonoxynol‐9 was used as a positive control for cytotoxicity. Viability was determined by measurement of formazan production in cell lysates. Results were expressed as the percentage of cellular viability attained compared to the negative, media‐only control, plus or minus the standard deviation (SD). Results were expressed as the percentage of cellular viability compared to the negative (medium‐only) control ± standard deviation (SD).

To investigate any potential antiviral activity of the same crude extracts, the luciferase assay was performed as previously described (Madan *et al*., 2007). Extracts were diluted to 50% concentration; then, a twofold serial dilution was performed. TZM‐bl cells (10^4^/well) were cultured in the presence of extracts for 72 h at 37 °C with a concentration of HIV‐1BaL optimized for infectivity. The extent of viral replication was determined by the quantification of luciferase on cell lysates (Promega, Madison, WI). Results were presented as the percentage of infection attained relative to the virus‐only control, plus or minus the standard error (SE). To determine the antiviral potency of the same crude extracts, they were titrated onto TZM‐bl cells (10^4^ cells well) in triplicate and cultured for 72 h with HIV‐1BaL using a concentration optimized for infectivity. Twofold serial dilutions of the extracts were prepared for testing. The extent of viral replication was determined by measuring luciferase activity in the cell lysates using a luciferase assay system (Promega, UK) as described by Wei *et al*. (2002). Results were expressed as the percentage of infection relative to the virus‐only control ± standard error (SE).

## Supporting information


**Table S1** Patents relevant to the commercialization of ^OS^GRFT.
