# Supplementary material for: Rice endosperm is cost‐effective for the production of recombinant griffithsin with potent activity against HIV
Source: Plant Biotechnol J. 2016 Jan 23;14(6):1427–37. doi: 10.1111/pbi.12507 (PMC4865440; doi:10.1111/pbi.12507)
Supplement: Supplementary file 1 — Table S1 Patents relevant to the commercialization of OSGRFT. [file PBI-14-1427-s001.docx]

| **Relevant claims** | **Utility for commercial development** |
| --- | --- |
| O’Keefe et al. (2011) Anti-viral griffithsin compounds, compositions, and methods of use. European Patent EP2314349. | |
| **Major claims:**   1. Use of an anti-viral polypeptide (GRFT) or an anti-viral fragment thereof comprising at least eight contiguous amino acids, optionally as part of a polypeptide conjugate, for the preparation of a medicament for inhibiting a SARS viral infection in a host. 2. Use of a cell transformed with a nucleic acid molecule that encodes an anti-viral polypeptide (GRFT) or an anti-viral fragment thereof comprising at least eight contiguous amino acids, for the preparation of a medicament for inhibiting a SARS viral infection in a host. 3. Use of a nucleic acid that encodes an anti-viral polypeptide (GRFT) or an anti-viral fragment thereof comprising at least eight contiguous amino acids, optionally in the form of a vector, for the preparation of a medicament for inhibiting a SARS viral infection in a host. 4. Use of an anti-GRFT antibody, for the preparation of a medicament for inhibiting a SARS viral infection in a host. 5. A method of removing a virus from a sample by contacting the sample with (a) an anti-viral polypeptide (GRFT) or an anti-viral fragment thereof comprising at least eight contiguous amino acids, optionally as part of a polypeptide conjugate, or (b) an antibody that binds to GRFT wherein the virus is SARS, and whereupon the virus is removed from the sample. | **Major constraints:**   1. **GRFT coding sequence.** Sequences claimed in the patent were used although a plant leader peptide was added. 2. **Cell-based production.** The patent lists a number of prospective host cells and although some animal cell lines are listed, microbes are preferred. The list does not include any plant cells or whole-plant systems but the patent claims are ‘not limited to’ the presented list. 3. **Vectors.** The patent lists several vectors for GRFT production but the vector used in the reported experiments is not on this list. 4. **Detection of GRFT.** The patent claims the use of rabbit anti-GRFT polyclonal antiserum, the same method we used. 5. **Purification based on the histidine tag.** Similar procedure used although the washing buffer composition was different. 6. **Method of use.** Provision of an anti-viral polypeptide and derivatives thereof, and broad uses thereof, including prophylactic and/or therapeutic applications against viruses. The principal application is for SARS infections but other viral targets are mentioned. |
| Boyd et al. (2011) Griffithsin, glycosylation-resistant griffithsin, and related conjugates, compositions, nucleic acids, vectors, host cells, methods of production and methods of use. US Patent US20110189105. | |
| **Major claims:**   1. An isolated and purified nucleic acid molecule that encodes a polypeptide (GRFT) comprising at least eight contiguous amino acids, wherein the at least eight contiguous amino acids have anti-viral activity, optionally as part of an encoded fusion protein. 2. An anti-viral polypeptide (GRFT) comprising at least eight contiguous amino acids, wherein the at least eight contiguous amino acids have anti-viral activity. 3. A method of inhibiting prophylactically or therapeutically a viral infection of a host, which method comprises administering to the host an effective amount of an anti-viral polypeptide or anti-viral polypeptide conjugate comprising at least eight contiguous amino acids of GRFT, wherein the at least eight contiguous amino acids have anti-viral activity and are optionally non-glycosylated, whereupon the viral infection is inhibited. 4. A method of inhibiting a viral infection of an animal, which method comprises transforming in vivo host cells with a nucleic acid molecule that encodes an anti-viral polypeptide comprising at least eight contiguous amino acids of GRFT, wherein the at least eight contiguous amino acids have anti-viral activity, to express an anti-viral polypeptide encoded by said nucleic acid molecule in vivo, whereupon the expression of said anti-viral polypeptide inhibits infection of the animal with a virus that can be inhibited by said anti-viral polypeptide. 5. A method of inhibiting a viral infection of an animal, which method comprises transforming host cells with a nucleic acid molecule that encodes an anti-viral polypeptide comprising at least eight contiguous amino acids of GRFT, wherein the at least eight contiguous amino acids have anti-viral activity, and placing said transformed host cells into or onto said animal so as to express in or on said animal an anti-viral polypeptide encoded by said nucleic acid molecule in vivo, whereupon the expression of said anti-viral polypeptide inhibits infection of the animal with a virus that can be inhibited by said anti-viral polypeptide. 6. An isolated and purified nucleic acid molecule that encodes a polypeptide comprising at least eight contiguous amino acids of GRFT, wherein the at least eight contiguous amino acids comprise amino acids 1-121 of GRFT which have been rendered glycosylation-resistant and wherein the at least eight contiguous amino acids have antiviral activity, optionally as part of an encoded fusion protein. 7. An antiviral polypeptide comprising at least eight contiguous amino acids of GRFT, wherein the at least eight contiguous amino acids comprise amino acids 1-121 of GRFT which have been rendered glycosylation-resistant and wherein the at least eight contiguous amino acids have antiviral activity. 8. An antibody that binds to GRFT. 9. An anti-GRFT antibody. 10. A method of inhibiting infection of a mammal with a virus, which method comprises: administering to the mammal an anti-GRFT antibody, or a composition comprising same, in an amount sufficient to induce in the mammal an immune response to the virus, which method optionally further comprises the prior, simultaneous or subsequent administration, by the same or a different route, of an antiviral agent or another agent that is efficacious in inducing an immune response to the virus, whereupon the infection of the mammal with the virus is inhibited. | **Major constraints:**   1. **GRFT coding sequence.** Sequences claimed in the patent were used although a plant leader peptide was added. 2. **Cell-based production.** The patent lists a number of prospective host cells and although some animal cell lines are listed, microbes are preferred. The list does not include any plant cells or whole-plant systems but the patent claims are ‘not limited to’ the presented list. 3. **Vectors.** The patent lists several vectors for GRFT production, but the vector used in the reported experiments is not on this list. 4. **Detection of GRFT.** The patent claims the use of rabbit anti-GRFT polyclonal antiserum, the same method we used. 5. **Purification based on the histidine tag.** Similar procedure used although the washing buffer composition was different. 6. **Method of use**. The methods in this patent are broader than the earlier patent. Uses include gene therapy as well as direct prophylactic and/or therapeutic applications of the peptide with and without glycosylation. |
| LiWang and Ionnis (2011) Bifunctional griffithsin analogs. US Patent US20110263485. | |
| **Major claims:**   1. An isolated chimeric polypeptide comprising a first portion comprising GRFT, amino acid residues 11 to 101 of GRFT, a substantial homologue of either one thereof, or an amino acid sequence that is at least about 80% identical to GRFT and a second portion selected from a gp41-binding protein, a CCR5-binding protein, a gp120-binding protein, GRFT, amino acid residues 11 to 101 of GRFT or a substantial homologue of any one thereof. 2. An isolated polypeptide (GRFT variants) or an amino acid sequence having at least 80% identity to them. 3. A composition comprising a first polypeptide comprising GRFT, amino acid residues 11 to 101 of GRFT, a substantial homologue of either one thereof or an amino acid sequence that is at least about 80% identical to GRFT and a second polypeptide selected from a gp41-binding protein, a CCR5-binding protein, a gp120-binding protein, a chimeric polypeptide comprising two different proteins selected from a gp41-binding protein, a CCR5-binding protein or a gp120-binding protein, or a substantial homologue of any one thereof. 4. An isolated chimeric polypeptide comprising a first portion comprising a gp41-binding protein and a second portion comprising a gp120-binding protein. | **Major constraints:**  None |
| O’Keefe et al. (2014) Monomeric griffithsin tandemers. World patent WO2014197650 | |
| **Major claims:**   1. A construct containing two or more monomeric griffithsin molecules, optionally joined by a linker. | **Major constraints:**  None |
